# Rare Earth Nanoprobes for Targeted Delineation of Triple Negative Breast Cancer and Enhancement of Radioimmunotherapy

**DOI:** 10.1002/advs.202309992

**Published:** 2024-05-22

**Authors:** Zi‐He Ming, Yong‐Qu Zhang, Liang Song, Min Chen, Lin‐Ling Lin, Yue‐Yang He, Wan‐Ling Liu, Yuan‐Yuan Zhu, Yun Zhang, Guo‐Jun Zhang

**Affiliations:** ^1^ Cancer Center and Department of Breast and Thyroid Surgery Xiang'an Hospital of Xiamen University School of Medicine Xiamen University Xiamen Fujian 361104 China; ^2^ State Key Laboratory of Structural Chemistry Fujian Institute of Research on the Structure of Matter Chinese Academy of Sciences Fuzhou Fujian 350000 China; ^3^ The Breast Center Yunnan Cancer Hospital The Third Affiliated Hospital of Kunming Medical University Beijing University Cancer Hospital Kunming Yunnan 650118 China; ^4^ Department of Breast Center Cancer Hospital of Shantou University Medical College Shantou Guangdong 515041 China; ^5^ Fujian Key Laboratory of Precision Diagnosis and Treatment in Breast Cancer Xiang'an Hospital of Xiamen University Xiamen Fujian 361102 China; ^6^ Xiamen Key Laboratory of Rare Earth Photoelectric Functional Materials Xiamen Institute of Rare Earth Materials Haixi Institute Chinese Academy of Sciences Xiamen Fujian 361021 China; ^7^ Xiamen Key Laboratory of Endocrine‐Related Cancer Precision Medicine Xiang'an Hospital of Xiamen University Xiamen Fujian 361102 China; ^8^ Xiamen Research Center of Clinical Medicine in Breast and Thyroid Cancers Xiamen Fujian 361102 China

**Keywords:** radioimmunotherapy, radiosensitization, rare‐earth nanoparticles, second near‐infrared‐fluorescence imaging, triple negative breast cancer

## Abstract

Radiotherapy demonstrates a synergistic effect with immunotherapy by inducing a transformation of “immune cold” tumors into “immune hot” tumors in triple negative breast cancer (TNBC). Nevertheless, the effectiveness of immunotherapy is constrained by low expression of tumor‐exposed antigens, inadequate inflammation, and insufficient tumor infiltrating lymphocyte (TILs). To address this predicament, novel lutecium‐based rare earth nanoparticles (RENPs) are synthesized with the aim of amplifying radiation effect and tumor immune response. The nanoprobe is characterized by neodymium‐based down‐conversion fluorescence, demonstrating robust photostability, biocompatibility, and targetability. The conjugation of RENPs with a CXCR4 targeted drug enables precise delineation of breast tumors using a near‐infrared imaging system and improves radiation efficacy via lutetium‐based radio‐sensitizer in vivo. Furthermore, the study shows a notable enhancement of immune response through the induction of immunogenic cell death and recruitment of TILs, resulting in the inhibition of tumor progression both in vitro and in vivo models following the administration of nanoparticles. Hence, the novel multifunctional nanoprobes incorporating various lanthanide elements offer the potential for imaging‐guided tumor delineation, radio‐sensitization, and immune activation post‐radiation, thus presenting an efficient radio‐immunotherapeutic approach for TNBC.

## Graphical abstract

The novel lutecium‐based rare earth nanoparticles RENPs offer the potential for imaging‐guided tumor delineation with CXCR4 target, radio‐sensitization with ROS production and tumor microenvironment immune activation with immunogenic cell death and immune cell recruitment. This multifunctional nanoprobe hold promises for enhancing the effectiveness of radioimmunotherapy in triple negative breast cancer synergistically.

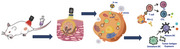

## 1 Introduction

In recent years, immune checkpoint inhibitors (ICIs), particularly programmed cell death protein 1/programmed cell death 1 ligand 1 (PD‐1/PD‐L1) inhibitors, have demonstrated significant efficacy in the clinical management of over 20 types of solid tumors, including breast cancer.^[^ ^1^ ^]^ The mechanism of PD‐1/PD‐L1 inhibitors involves the activation of T cells, restoration of depleted T cell function, and augmentation of T cell‐mediated anti‐tumor response in metastatic and recurrent triple negative breast cancer (TNBC).^[^ ^2^ ^]^ Despite these advancements, the response rate to PD‐1/PD‐L1 inhibitor monotherapy remains modest, typically ranging from 20–30%.^[^ ^3^ ^]^ This is primarily caused by immunosuppressive tumor microenvironment (TME), inadequate tumor infiltrating lymphocytes (TILs), varying phenotypes of macrophages in TME,^[^ ^4^ ^]^ lack of PD‐L1 expression, and weak tumor‐specific antigens.^[^ ^5^ ^]^

It is widely acknowledged that radiotherapy (RT) can improve long‐term survival in breast cancer patients by mitigating locoregional recurrence.^[^ ^6^ ^]^ In addition, previous researches have demonstrated that RT has the capacity to trigger immunogenic cell death (ICD), promote dendritic cells (DCs) maturation.^[^ ^7^ ^]^ RT can recruit TILs and broaden the T‐cell receptor repertoire within tumors specifically.^[^ ^8^ ^]^ Furthermore, it has been observed that RT increases the expression of PD‐L1 on tumor cells,^[^ ^9^ ^]^ leading to the enhancement of PD‐L1 inhibitors’ efficacy and improvement of survival outcomes for cancer patients. A number of clinical trials are investigating the synergistic effects of RT and immune checkpoint therapy (ICT), also known as radioimmunotherapy,^[^ ^10^ ^]^ with a remission rate up to 53.3% in lung cancers receiving first‐line treatment.^[^ ^11^ ^]^ Despite the noticeable increase in response rate, there remains significant space in optimizing therapeutic outcomes. It is anticipated that the efficacy of ionizing radiation combined with chemotherapy may be augmented by radiosensitization. In order to enhance therapeutic efficacy and extend survival time, a novel approach is required to enhance RT sensitization and stimulate tumor immunity in TNBC patients ultimately.

Increasing RT dosages may enhance locoregional control and therapeutic efficacy in TNBC, but excessive radiation exposure can lead to significant normal tissues damages and severe side effects.^[^ ^12^ ^]^ Consequently, augmenting of inherent radiosensitivity through nanoparticle‐mediated approach is anticipated to bolster regional control without necessarily escalating dosages. This has motivated the exploration of a novel strategy to enhance the radiosensitivity and identify tumor localization concurrently, addressing the issue of suboptimal therapeutic outcomes in TNBC patients. In order to attain the objectives in vivo, the utilization of rare earth nanoparticles as a visualized and targeted radiosensitizer may be a viable strategy.

Rare earth nanoparticles are often utilized in the construction of multifunctional probes due to their distinctive characteristics in probe design.^[^ ^13^ ^]^ For instance, probes with a core‐shell structure can be tailored for RT localization and sensitization. The core, when doped with luminescent element such as neodymium (Nd), exhibits excellent near‐infrared (NIR) imaging properties. Lutetium (Lu), the lanthanide element with the largest atomic number, possesses a large X‐ray attenuation coefficient and absorbs energy produced by X‐rays. Following irradiation, Lu will emit secondary electrons such as photoelectrons, Auger electron, and Compton electrons.^[^ ^14^ ^]^ As a result, water molecules will generate the reactive oxygen species (ROS), leading to irreversible DNA damage and tumor apoptosis ultimately.^[^ ^15^ ^]^ Moreover, an ideal target of recognizing tumor cells specifically is essential for the precise guidance of tumors. The CXCR4 protein, a crucial G protein coupled receptor expressed on the cell membrane, exhibits high expression in TNBC compared to other subtypes of breast cancer, and is correlated with unfavorable prognostic outcomes.^[^ ^16^ ^]^ Therefore, CXCR4 represents an optimal guidance target in TNBC and its small molecule inhibitor AMD3100 approved by FDA in 2008 is chosen to link to the nanoparticles.

**Scheme 1:**
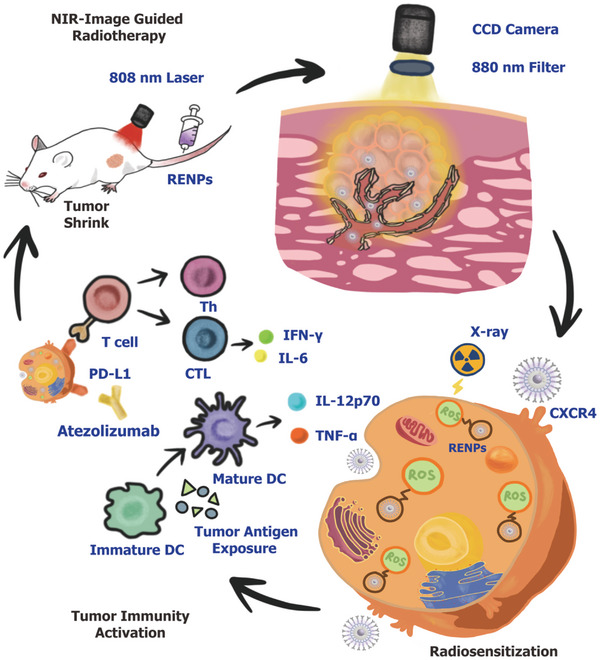
Schematic illustration of the RENPs for precise guidance and sensitization of radiotherapy in synergism with ICT.

In this research, a novel nanoprobe was developed utilizing core‐shell structure of NaNdF_4_:Yb@ NaLuF_4_ and surface modification with DSPE‐PEG_2000_‐COOH and AMD3100 (RENPs). The study shows that RENPs can target TNBC tumors, induce ROS production, and enhance the radiosensitivity of tumors ultimately. Additionally, RENPs promote tumor‐specific antigen presentation, immune cell recruitment, immunogenic cell death and inhibit tumor growth when combined with ICT in synergistic manner (**Scheme** **1**).

## 2 Results

### 2.1 Preparation and Characterization of RENPs

**Figure 1:**
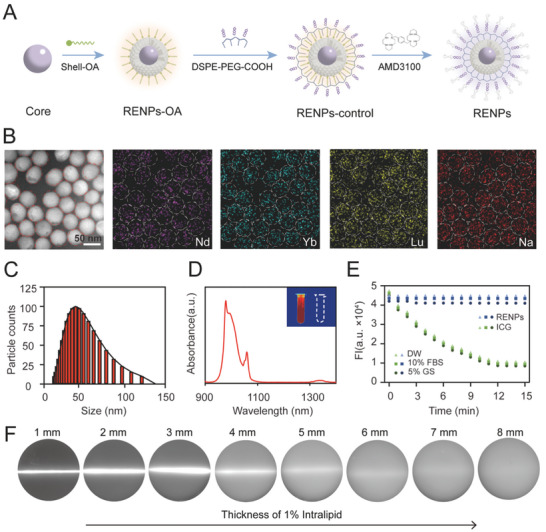
Characterization of RENPs. A) Schematic diagram of probe synthesis. B) HAADF‐STEM image of core‐shell structure in RENPs and corresponding Nd/Yb/Lu/Na elemental mapping. C) DLS analysis of hydrodynamic size distribution of RENPs‐OA. D) Fluorescence emission spectra of RENPs. The left insert image was RENPs while the right one is DW of NIR‐II fluorescence imaging. E) Stability of RENPs and ICG in different solution including DW, 10% FBS and 5% GS under continuous laser irradiation. F) NIR‐II fluorescence images of RENPs following the increase of the thickness of 1% intralipid. Scale bar: 50 nm.

In order to obtain the multi‐functional nanoprobes (NIR imaging/RT sensitization/TME activation), we synthesized core‐shell structure rare earth nanometer material through layer‐by‐layer epitaxial growth strategy. The rational design of RENPs was presented in **Figure** **1**A. The core particle NaNdF_4_:Yb was synthesized by high temperature co‐precipitation method, shell structure NaLuF_4_ was subsequently coated by epitaxial growth method, and CXCR4 inhibitor AMD3100 was conjugated after the surface modification of DSPE‐PEG_2000_‐COOH to obtain the multi‐functional molecular probe RENPs.

RENPs were first explored in different input ratios and then verified its successful synthesis by several characterization experiments. First, high angle annular dark field scanning transmission electron microscope (HAADF‐STEM) was used to observe the morphology of the probe and estimate its particle size roughly, the results showed that the different doping ratios of Nd to Yb in NaNdF_4_:Yb core nanoparticles were different in morphology (Figure S1A, Supporting Information). The best ratio of Yb/Nd is 15% because the particles have uniform oval shapes and better fluorescence (Figure S1B, Supporting Information). The successfully synthesized core NaNdF_4_:Yb and core‐shell particles NaNdF_4_:Yb@NaLuF_4_ were clear and transparent lavender liquid (Figure S1C, Supporting Information). Energy‐dispersive X‐ray spectroscopy elemental maps and spectra of nanoparticles revealed the homogeneous distribution of Nd, Yb, Lu, and Na in RENPs‐OA (Figure 1B). Dynamic light scattering (DLS) results showed the average particle size of RENPs‐OA was ≈40 nm (Figure 1C). In order to achieve better biocompatibility, RENPs‐OA was coated with DSPE‐PEG_2000_‐COOH (RENPs‐control) and AMD3100 (RENPs). The hydrated particle size of RENPs‐control and RENPs increased gradually, which is shown by DLS (Figure S1E, Supporting Information). The structures of different stages of RENPs (RENPs‐OA, oil phase solution; RENPs‐control, aqueous solution without AMD3100; RENPs, aqueous solution with AMD3100) was also analyzed by Fourier‐transform infrared (FTIR) (Figure S1F, Supporting Information) and ultraviolet spectrophotometer (Figure S1G, Supporting Information) to verify that the components were connected successfully, including DSPE‐PEG_2000_‐COOH and AMD3100.

The probes also have excellent fluorescence performance. Under the excitation laser of 808 nm, the fluorescence spectrometer detected the high intensity NIR fluorescence of the molecular probe at 979, 1065, and 1330 nm (Figure 1D). Also, the fluorescence intensity improved with the increase of RENPs concentration without aggregation quenching (Figure S1D, Supporting Information). It was, however, only a slight loss of fluorescence in the progress of probe synthesis because the probe has changed its phase from oil to water (Figure S1H, Supporting Information). The stability of fluorescence plays an important role in defining tumor location in case of quenching under the excitation of laser. We tested the fluorescence intensity of RENPs under continuous laser exposure, compared with another NIR common dye indocyanine green (ICG). The results showed that both ICG and RENPs were stable in double distilled water (DW), 10% fetal bovine serum (FBS), and 5% glucose solution (GS). However, the fluorescence intensity dropped sharply in ICG while RENPs remained unchanged under continuous 808 nm laser exposure in 15 mins (Figure 1E). Last but not least, the penetration is the critical characteristic of RENPs because it is supposed to guide the tumor localization under the skin. With the cover of various thickness of 1% intralipid (each group is 1 mm thicker), we found RENPs showed deep penetration of 8 mm in NIR‐II imaging (Figure 1F).

### 2.2 In vitro Uptake of RENPs

**Figure 2:**
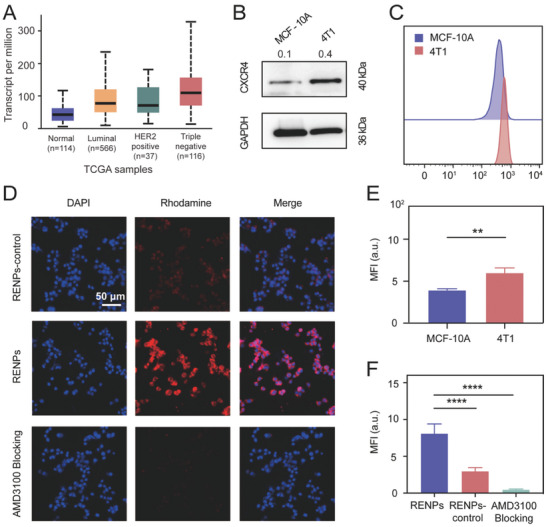
In vitro targeted aggregation of RENPs. A) Expression of CXCR4 in BRCA based on breast cancer subclasses in TCGA samples. B) Western blot analysis of CXCR4 expression level in different cell lines. The numbers above the black box are quantitative data, which represent CXCR4 expression/GAPDH expression. C) Flow cytometry of MCF‐10A and 4T1 cells incubated with RENPs and E) corresponding quantified of mean fluorescence intensities (MFI) (*n* = 3). D) Representative fluorescence microscope scanning of 4T1 cell crawling after incubated with RENPs‐control and RENPs, one group incubated with AMD3100 ahead to block CXCR4 and F) corresponding mean MFI (*n* = 5). Scale bar: 50 µm. **P < 0.01, ****P < 0.0001.

In order to clarify the expression of CXCR4 in different subtypes of breast cancer, we searched the samples from the cancer genome atlas (TCGA) database. The result showed that the expression of CXCR4 in TNBC is the highest (**Figure** **2**A). Western blot results also presented higher expression of CXCR4 in TNBC cell line 4T1 than normal mammary epithelial cell line MCF‐10A (Figure 2B).

Before investigating the target effect of RENPs, the endocytosis was evaluated through 4T1 cell line. Time‐dependent and concentration‐dependent intercellular uptake of RENPs in 4T1 were first evaluated by flow cytometry assay so that we can choose appropriate time and concentration to incubate cells in the experiments afterwards (Figure S2A,B, Supporting Information). The fluorescent microscopy scanning of cell crawling also showed the intake of nanoparticles in 4T1 cells (Figure S2C,D, Supporting Information) in a more intuitive way.

After determining the appropriate incubation time and concentration (3 h, 50 µg mL^−1^), we investigated the target effect of RENPs in vitro. Flow cytometry assay showed higher absorption level of RENPs in 4T1 than MCF‐10A (3.957 ± 0.081 *vs* 6.010 ± 0.327, *P* < 0.01) (Figure 2C,E), this result indicated that the nanoparticles can identify tumor cells in subcellular level. Besides, we applied two kinds of probes with or without target element AMD3100 (RENPs‐control and RENPs) in the same cell line to reverify the target property of probes (5.407 ± 0.6217 *vs* 10.90 ± 0.4067, *P* < 0.01) (Figure S3, Supporting Information). The cell crawling results confirmed the CXCR4 target effect of RENPs as well (Figure 2D). Compared with the group treated with RENPs (with AMD3100), the group treated with RENPs‐control (without AMD3100) had a relative low level of fluorescence intensity because of the nonspecific absorption in cells (8.096 ± 0.5831 *vs* 2.980 ± 0.2180, *P* < 0.0001). In contrast, in the blocking group, CXCR4 was blocked by the CXCR4 inhibitor AMD3100 previously, so the mean fluorescence intensity of group pretreated with AMD3100 was significantly lower because of the site preemption in CXCR4 (8.096 ± 0.5831 *vs* 0.4736 ± 0.0442, *P* < 0.0001) (Figure 2F). In this blocking group, it didn't exhibit nonspecific binding of RENPs compared to RENPs‐control group.

### 2.3 In vitro RT Sensitization and DCs Maturation by RENPs Plus RT

**Figure 3:**
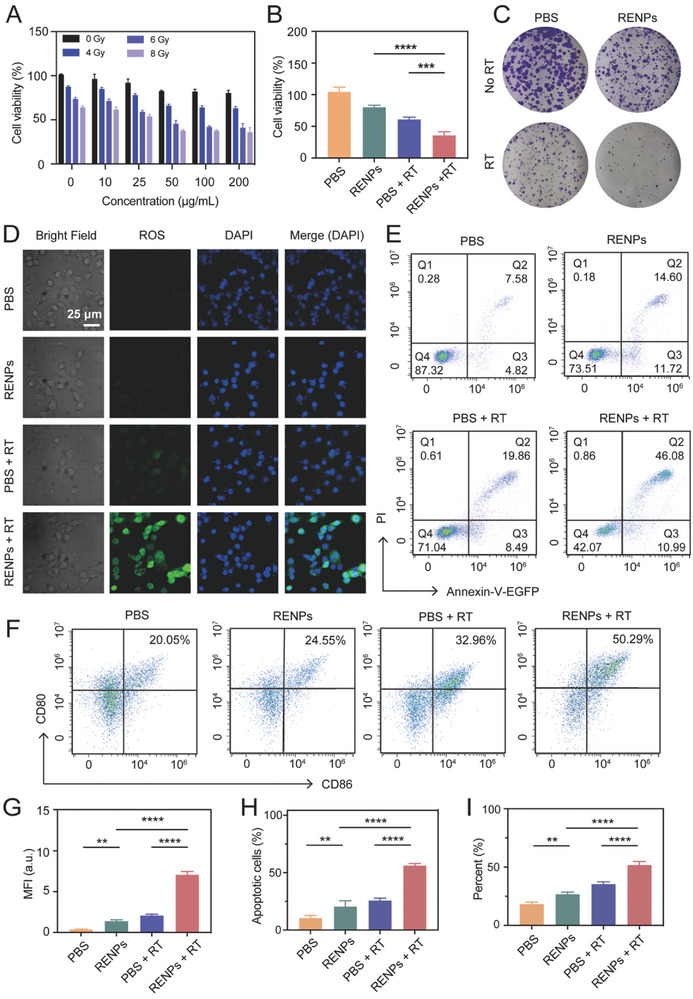
RT sensitization and DCs maturation by RENPs plus RT in vitro. A) Viability of 4T1 cells incubated with RENPs in different concentration under different dose of X‐ray irradiation and B) corresponding quantified of cell viability treated with 50 µg mL^−1^ RENPs under 6 Gy. C) Colony formation assay of 4T1 cells after various treatments. D) Fluorescent images of intracellular ROS generation in 4T1 cells after different treatments. E) Flow cytometry analysis of apoptosis rates in 4T1 cells after different treatments. F) Flow cytometry analysis of surface expression of CD80^+^ CD86^+^ on DCs after different treatments. G) Statistical analysis of mean fluorescence intensity in (D). H) Statistical analysis of apoptotic cells in (E). I) Statistical analysis of mature DCs ratio in (F). Data were given as mean ± S.D. Scale bar: 25 µm. **P < 0.01, ***P < 0.001, ****P < 0.0001, *n* = 3.

During the process of RT sensitization, energy of X‐ray will be absorbed by highest‐Z lanthanide element Lu to release secondary electrons. These electrons can be captured by H_2_O molecules in tumor sites and generate ROS. To examine the RT sensitization efficacy of RENPs, CCK8 assay was conducted to evaluate the RENPs antiproliferative effects under different doses of X‐ray irradiation (0, 4, 6, and 8 Gy) and different concentration of RENPs. The cytotoxicity appeared gradually with the improvement of irradiation dose and drug concentration (**Figure** **3**A). In the groups treated without irradiation (PBS and RENPs group), RENPs group showed a little cytotoxicity because of AMD3100, a CXCR4 inhibitor which can treat TNBC. And the exact treatment effect will be mentioned in the safety experiments afterwards. As is shown in Figure 3B, 50 µg mL^−1^ RENPs plus with 6 Gy X‐ray irradiation induced ≈65% death of 4T1 cells while 40% in PBS + RT group (Cell viability: 61 ± 2.082 *vs* 36 ± 3.215, *P* < 0.001), indicating that the RENPs‐mediated radiotherapy could kill tumor cells more effectively.

The colony formation assay is the golden standard for evaluation of radiosensitivity in vitro because only a part of cells maintained the replicative potential to form colonies continuously after radiotherapy. Herein, we set four groups including PBS, RENPs, PBS + RT and RENPs + RT in this assay (Figure 3C). Obviously, in the RENPs + RT group, only a few viable colonies (less than 50 cells) were formed, which couldn't be scored for survival. In the irradiated groups, the tumor cells were dispersed and showed a small number of cell colonies when incubated with RENPs, suggesting the continuously proliferated ability of treated cells was impaired (Figure S4, Supporting Information). By contrast, the nonirradiated PBS groups exhibited abundant cell colonies, which showed obvious large purple cell clumps after crystal violet staining. So, the colony formation assay certificated that RENPs sensitized radiotherapy could effectively prevent cell proliferation in a long term.

To further investigate the mechanism of cell death after the treatment of RENPs plus RT, we detected the intracellular ROS production in 4T1 cells (Figure 3D). After incubating with ROS detecting fluorescent probe DCFH‐DA, irradiated 4T1 cells incubated with RENPs exhibited significantly high‐intense intracellular fluorescence than the cells incubated with PBS (2.076 ± 0.09455 *vs* 7.068 ± 0.2235, *P* < 0.0001), which meant more ROS was produced. Meanwhile, the nonirradiated PBS and RENPs groups exhibited negligible fluorescence signals (0.3687 ± 0.01596 *vs* 1.386 ± 0.095, *P* < 0.01) (Figure 3G). This result indicated the generation of ROS may contribute the death of tumor cells after combination treatment.

RENPs‐mediated RT sensitization in 4T1 cells was also evaluated by an apoptosis assay to identify cell status after the treatment (Figure 3E). After coincubation with annexin V‐fluorescein isothiocyanate (FITC) and propidium iodide (PI), early apoptotic cells were only stained by annexin V‐FITC, while late apoptotic or necrotic cells were co‐stained with two dyes. Flow cytometry showed the apoptosis rate of normal cells was less than 10%. However, significant apoptosis rate (57.07%) was elicited in RENPs + RT group, compared with other groups (26.32% in RENPs group and 28.35% in PBS + RT group) (Figure 3H).

To conclude, the RENPs‐treated TNBC cells induced remarkably lower cell viability (55.67%), higher cell apoptosis (57.07%) and higher level of ROS (22 folds higher than control group) after X‐ray irradiation compared to other groups. All these results suggested that the RENPs elicited a meaningfully RT sensitization effect in vitro.

As the main type of antigen‐presenting cells (APCs), DCs can present tumor antigens which released from dead tumor cells and initiate T cell‐mediated adaptive immune response to activate anti‐tumor immunity. This process translates immature DCs to mature DCs, with the up‐regulated costimulatory molecules (CD80 and CD86) and secretion of cytokines. Immature DCs extracted from naïve mouse bone marrow and 4T1 cells treated with RENPs + RT were first cocultured in the transwell plate, then we investigated the maturation ratio of DCs to confirm the immune response triggered by tumor antigen exposure (Figure 3F). After incubating with the co‐biomarker of CD11, flow cytometry analysis indicated that the proportion of mature DCs in the RENPs + RT group increased to 50.29%, which was significantly higher than that in PBS group (20.05%), RENPs group (24.55%) and PBS + RT group (32.96%). The results showed that 4T1 cells pretreated with RENPs + RT can promote maturation of DCs and induce sustained immune activation apparently (Figure 3I).

### 2.4 Targeted Aggregation and RT Sensitization of RENPs in vivo

**Figure 4:**
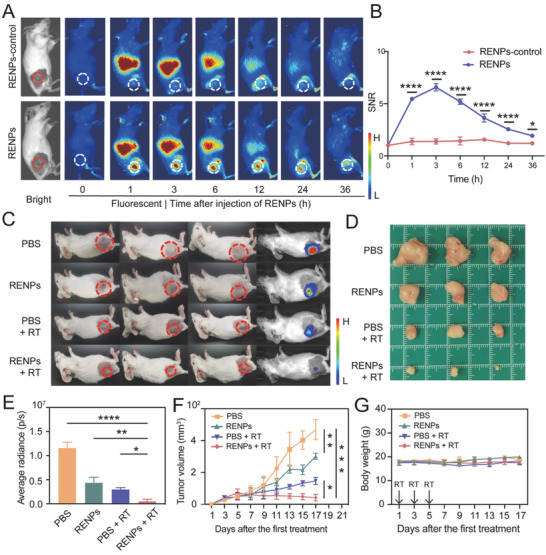
Target effect and RT sensitization effect of RENPs in subcutaneous tumor bearing mouse. A) Bright images and NIR fluorescence images of representative mice injected with RENPs‐control and RENPs at different time points post‐injection. The red/white dotted ellipses indicate the tumor position. B) The SNRs of tumor‐bearing mice injected RENPs‐control and RENPs at different time points. (*n* = 3) C,D) Photographs of mice and excised tumor after different treatments. E) Average radiance of tumor bioluminescence after different treatments. F) Tumor volume of transplanted tumor after different treatments. G) Body weight of mice after different treatments. Data are presented as the mean ± SD (*n* = 3), *P < 0.05, **P < 0.01, ***P < 0.001, ****P < 0.0001.

The tumor‐targeting ability of RENPs in vivo was evaluated in the subcutaneous 4T1 tumor‐bearing BALB/c mice after intravenous injection of RENPs with and without AMD3100 functionalization (RENPs‐control and RENPs) (**Figure** **4**A). The NIR bioimaging result showed the signal to noise ratios (SNRs) increased gradually over time in the RENPs group (6.576 ± 0.347), while the RENPs‐control group did not change obviously after injection (1.384 ± 0.256). Simultaneously, the maximum SNRs of the RENPs group were remarkably higher than those in the RENPs‐control group in 3 h (6.576 ± 0.347 *vs* 1.384 ± 0.256, *P* < 0.001, Figure 4B). The fluorescent signal of targeted probe group is ≈fivefolds than untargeted group, suggesting the excellent tumor targeting ability of RENPs. Furthermore, tumors and organs were collected to obtain the *ex vivo* fluorescence images, the results showed that RENPs unevenly distributed in different tissues. RENPs mainly accumulated in the spleen, liver and bone which were regarded as the nonspecific phagocytosis of reticuloendothelial system (RES). Besides, RENPs also concentrated at the tumor because of the target ability (Figure S5, Supporting Information). Heart, kidney, muscle, and skin exhibited little fluorescence, which meant there existed little probes in these organs.

In the cell experiments, we verified Lu‐based nanoprobes could notably increase ROS production and cell death in cancer after administration of RENPs plus with radiation. It is assumed that the mice intravenously injected with RENPs and treated with subsequent radiotherapy can achieve the tumor elimination similarly. Female BALB/c mice were inoculated with 4T1 tumors in the right leg, then randomly grouped and administrated with various treatments, including PBS, RENPs, PBS + RT, and RENPs + RT. As shown in Figure 4C, the nonirradiated group treated with PBS exhibited no tumor growth inhibition as control group. In contrast, all X‐ray irradiation groups (6 Gy, 3 times, inject every other day) showed significant tumor regression. RENPs‐treated mice exhibited efficient RT sensitization compared with the mice that only received X‐ray irradiation, and one tumor even completely disappeared (Figure 4D). When the mouse tumors were excised on day 17 after treatment, the tumor volume was assessed by the images of bioluminescence (Figure 4C,E). It was evident that the tumor volume was smaller in the radiotherapy sensitized group compared with other groups (Figure 4F). Moreover, no obvious difference in bodyweight was noted among the various groups, indicating that RENPs‐based radiotherapy sensitization did not cause great damage in mice (Figure 4G). Overall, RENPs plus X‐ray irradiation treatment could confer an effective inhibition of tumors with limited adverse effects.

### 2.5 Activation of Anti‐Tumor Immunity by RENPs Plus RT

**Figure 5:**
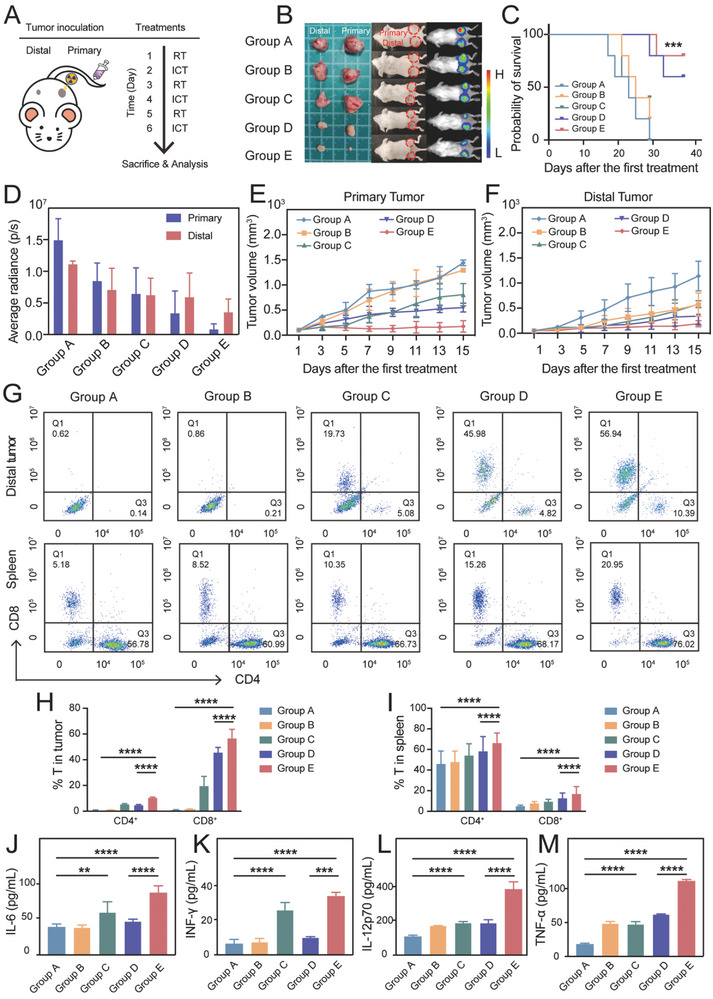
The antitumor immune response induced by combination therapy. A) Design of combination therapy in mouse. Group A received PBS. Group B received PBS + ICT. Group C received RENPs + ICT. Group D received PBS + RT + ICT. Group E received RENPs + RT + ICT. B) Images of mice and excised tumor after different treatments. C) Kaplan‐Meier analysis in survival of different groups. D) Average radiance of tumor bioluminescence after different treatments. E) Primary tumor volume after different treatments. F) Distal tumor volume after different treatments. G) CD8^+^ and CD4^+^ T cells proportions in distal tumors and spleen evaluated by flow cytometry. H,I) Quantitative analysis of mean fluorescence intensity in (G). J–M) Concentration of IL‐6, IFN‐γ, IL‐12p70, and TNF‐α cytokines in mouse serum tested by ELISAs. Data are presented as the mean ± SD (*n* = 3), *P < 0.05, **P < 0.01, ***P < 0.001, ****P < 0.0001.

To examine the anti‐tumor immune response activated by RENPs plus with X‐ray irradiation, two subcutaneous 4T1 tumors with different cell densities were concurrently established on the bilateral flank of female BALB/c mice, designated as primary (right) and distal (left) tumors. The primary tumor was treated with direct X‐ray radiation plus with ICT and the distal tumor was treated with systematic ICT. On day 10 after tumor inoculation, primary tumors (274.7 ± 33.4 mm^3^) were larger than distal tumors (123.4 ± 21.8 mm^3^), then we start combination treatment. Mice were randomly grouped and administrated with various treatments, including Group A (control, PBS only), Group B (PBS + ICT), Group C (RENPs + ICT), Group D (PBS + RT + ICT), and Group E (RENPs + RT + ICT). The groups treated with RENPs (Group C and Group E) were injected through tail vein every other day (Day 1, 3, 5), the group treated with RT (Group D and Group E) were irradiated with 6 Gy every other day (Day 1, 3, 5), and the groups treated with ICT (Group B, C, D, and E) were injected atezolizumab through abdominal cavity every other day (Day 2, 4, 6) (**Figure** **5**A). After the combination treatment of RT and ICT, the mice were sacrificed and analyzed, the different efficacy after treatments was shown in Figure 5B,C. Compared with Group A, Group B and Group C had a limited primary tumor inhibition owing to the single effect of ICT, but this effect is too minor to cure the cancer. Among them, the tumor volume in the RENPs treatment group was smaller than that in the PBS treatment group, which was also confirmed by cell experiments. Apparently, the primary and distal tumors in Group D and E shrank significantly due to the effect of RT. In primary tumors, a combination of RENPs and radioimmunotherapy inhibited the tumor growth more efficiently in Group E, which may attribute to the radiotherapy sensitization of RENPs. The bioluminescence imaging in tumor confirmed this result in a more precise and reliable way (Figure 5D,E).

Radiation can cause the abscopal effect, that is, the non‐irradiated distal tumor lesions also exhibit shrinkage. To investigate whether these treatments could influence the growth of unirradiated tumors, we evaluated the variations of non‐irradiated distal tumor volume (Figure 5F). Compared with other groups, Group D and Group E have significant inhibitory effect on distal tumors, and the inhibition of tumor may attribute to the activation of immune system in vivo. The result indicates that under the effect of X‐ray irradiation, the abscopal effect can reduce tumor volumes in the non‐irradiated field. In Group E, the abscopal effect seems to be stronger because of the administration of RENPs, we assumed that the RT sensitization could exaggerate the immune response in radiation groups. Additionally, the survival rate of mice was monitored after different treatments using 1500 mm^3^ tumor volume as the endpoint criteria. We found Group E displayed a greater survival benefit than Group D, this result also indicated the stronger activation of immunity by radiosensitization (Figure 5C). The body weight of the mice in the different groups was not affected substantially (Figure S6, Supporting Information). Overall, RENPs together with the radioimmunotherapy could confer an effective inhibition of distal tumors with limited adverse effects. When it comes to clinic, we can envision that this kind of strategy can be used to treat recurrent and metastatic TNBC patients especially in those who lose the surgical opportunity.

To figure out the exact activation mechanism of anti‐tumor immunity, we collected the tumors, spleen and serum from mice for further analysis. The crucial marker of immune response in tumor is the proportion of immune cells including cytotoxic T cells (CD3^+^CD8^+^) and helper T cells (CD3^+^CD4^+^), which could be evaluated by counting immune cells extracted from tumor. As shown in Figure 5G, flow cytometry was used to assess the ratio of CD8^+^ and CD4^+^ T cells in the distal tumor and spleen. After co‐staining with CD3, the T cells were catalogued by CD4 and CD8 to determine the state of antitumor immune activation. It turned out that no big difference was observed in the amount of CD4^+^ T and CD8^+^ T cells among Group A&B in distal tumor. Only a slight increase in CD8^+^ T cells of the Group C (19.73% ± 0.9% in distal tumors) was observed, possibly because RENPs triggered a mild immune response by its target drug AMD3100. Among them, Group D and Group E, which received radioimmunotherapy, showed a significant increase in the proportion of T cells in the distal tumor. Compared with Group D, we found an enormous elevation in both CD8^+^ T cell and CD4^+^ T cell fractions of distal tumor and spleen in Group E, suggesting that RENPs relieved immune tolerance and promoted an effective antitumor immune response (Figure 5H,I). Cytokines changes were also analyzed by enzyme‐linked immunosorbent assays (ELISAs) after 20 days of different treatments. Interleukin‐6(IL‐6) is an important inflammatory indicator, the increase of IL‐6 in Group C and Group E may be caused by the transient pro‐inflammatory effects of RENPs (Figure 5J). This has also been verified in the safety blood biochemical results. Interferon gamma (IFN‐γ) is a crucial nexus for controlling PD‐1‐mediated tumor infiltration by T cells,^[^ ^17^ ^]^ so the increasement of IFN‐γ in Group C and Group E can expand the effect of anti‐PD‐L1 therapy in mice (Figure 5K). Interleukin‐12p70 (IL‐12p70) is primarily produced by DCs, macrophages, B lymphocytes, and other APCs, it also induces the generation of specific CTL cells.^[^ ^18^ ^]^ The appreciably increasement of IL‐12p70 in Group E also confirms the previous results of the maturation of DCs and elevation of CTLs in other side (Figure 5L). Tumor necrosis factor‐α (TNF‐α), as a key regulator of innate immunity, has a direct killing effect on tumor cells. Group E displayed a dramatically increase in TNF‐α, which indicated the strong stimulation of inflammatory response (Figure 5M). Compared with the untreated group, the secretion of all these cytokines was significantly elevated in Group E, demonstrating that RENPs combined with radioimmunotherapy induced a powerful systematic immune response.

**Figure 6:**
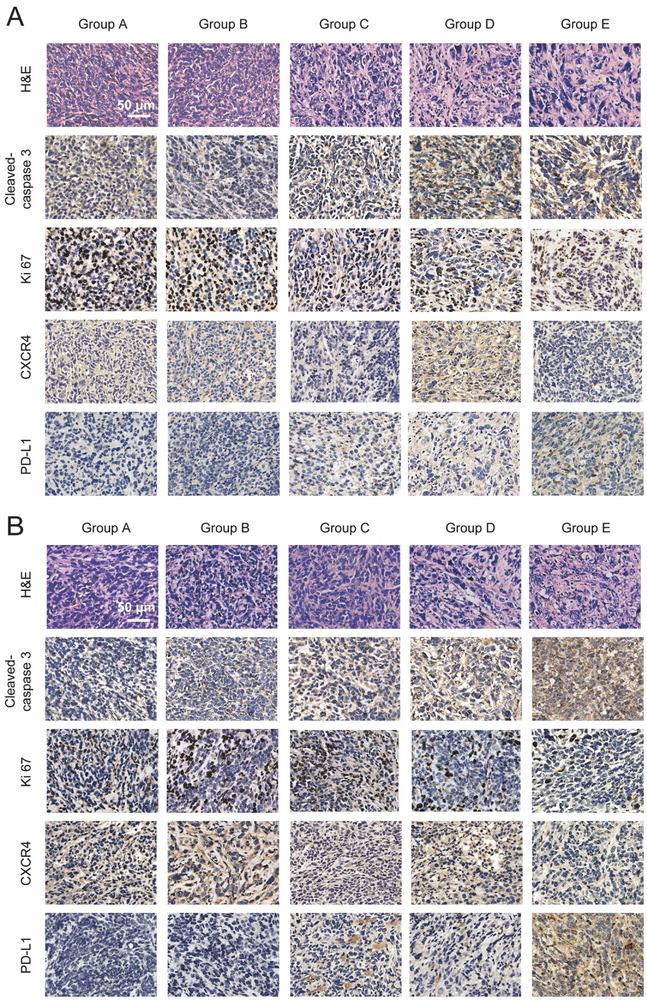
H&E staining, cleaved‐caspase 3, Ki 67, CXCR4 and PD‐L1 IHC staining images of tumor slices after various treatments. A) Primary tumor slices of Cleaved‐caspase 3, Ki 67, CXCR4, and PD‐L1 expression on tumor cells. B) Distal tumor slices of Cleaved‐caspase 3, Ki 67, CXCR4, and PD‐L1 expression on tumor cells. Scale bar: 50 µm. Group A received PBS. Group B received PBS + ICT. Group C received RENPs + ICT. Group D received PBS + RT + ICT. Group E received RENPs + RT + ICT.

Subsequent hematoxylin and eosin (H&E) staining of bilateral tumors revealed minimal nuclear staining due to tumor destruction following radiosensitization combined with immunotherapy (**Figure** **6**; Figure S7, Supporting Information). Among them, apoptosis‐related factors and tumor‐related markers in different tumor tissues were analyzed by immunohistochemical (IHC) staining. In clinical practice, Ki67 is mainly used to label cells in the proliferation cycle, which is an antigen related to mitosis. Ki67‐IHC stained tumor sections indicated that Group E exhibited less highly proliferative tumor cells compared with the other groups. In addition, cleaved‐caspase 3, a major marker of apoptosis, was significantly higher in Group E than in other groups. And this factor may be responsible for restraining cancer cell proliferation and inducing cell apoptosis in Group E. As a result, RENPs can activate anti‐tumor immune response under the synergistic effect of RT and ICT. In addition, we investigate the CXCR4 expression and PD‐L1 expression of tumor tissues after different treatments. As specific tumor marker, CXCR4 was significantly down‐regulated in Group C&E, which may be attributed to the CXCR4 inhibitor AMD3100. It is worth noting that PD‐L1 is an important marker of tumor cells, and its high expression on the surface of tumor cells can produce a higher response rate in anti‐PD‐L1 immunotherapy. The PD‐L1 expression can increase after the treatment of radiotherapy, as a result, the group D&E treated with RT showed a slight increase of PD‐L1 expression.

### 2.6 Biocompatibility of RENPs in vitro and in vivo

To evaluate biosafety of nanoparticles, CCK‐8 assay was first conducted to evaluate the toxicity and treatment effect of RENPs. MCF‐10A group and 4T1 group were compared after treatment of RENPs, negligible cytotoxicity was found in MCF‐10A which meant RENPs exhibit little damage in normal breast cell line. And RENPs can inhibit the 4T1 tumor cell growth, however, the effect is hard to kill most of tumor cells (Figure S8A, Supporting Information). Second, we verified the reason of cell death is whether the killing effect of AMD3100 or the toxicity of probe itself. With the increase of concentration, the anti‐tumor effect of AMD3100 in RENPs is more obvious (Figure S8B, Supporting Information). These results showed that RENPs had great target property and a slight treatment effect, making it a potential probe to guide the area of radiotherapy for eliminating breast cancer.

To investigate whether we can inject RENPs through mice tail vein, we tested the hemolysis of probes (Figure S9, Supporting Information). Additionally, the body weight of mice, histological analysis for major organs and serum biochemical markers including lactate dehydrogenase, creatine kinase, alanine aminotransferase, aspartate transaminase and creatinine were investigated. Fortunately, there was no influence on the body weights, no histopathological changes in major organs, and no significant difference in serum biochemistry. There is only slight elevation of ALT and AST on the first day, demonstrating that these treatments did not induce obvious systemic adverse effects (Figures S10 and S11, Supporting Information).

## 3 Discussion

Advanced TNBC patients often have rapid tumor progression, show resistance to multiple therapies, and associate with lower survival rates.^[^ ^19^ ^]^ While both RT and ICT show promising improvement of local disease control and prolong survival, only a small proportion of TNBC patients can benefit from ICT due to the immunosuppressive TME.^[^ ^20^ ^]^ Previous studies have demonstrated that RT can induce ICD, activate TME and increase PD‐L1 expression in tumors, thus improving the antitumor effect of ICT.^[^ ^21^ ^]^ Hence, it is rational to activate the immune response, thereby enhancing the efficacy of ICT. Recently, a phase II clinical trial was conducted to evaluate the efficacy of radioimmunotherapy in metastatic TNBC.^[^ ^22^ ^]^ Additionally, another phase II clinical trial showed an increase in survival rate from 6.7% to 53.3% with the use of radioimmunotherapy in lung cancer.^[^ ^11^ ^]^ Although the study has demonstrated some improvement in patient survival, there still remains significant space for enhancing therapeutic responses, such as improving radiosensitization. Therefore, introducing new multifunctional nanoparticles fabricated with targeting elements, might greatly sensitize tumors to X‐ray irradiation and further improve the effects of ICT in the meantime.^[^ ^23^ ^]^

In this study, we have developed a novel Lu‐based core‐shell nanoparticles that are conjugated with the CXCR4 inhibitor AMD3100. This novel nanoparticle aims to enhance RT sensitivity and stimulate tumor immunity, optimize the therapeutic effects ultimately. The nanoparticle was synthesized using the high‐temperature solid‐state method to construct a downshifting luminescence rare earth nanoprobe. The optimal ratio of Nd and Yb in the core construction was determined through successful screening, and the Lu in the shell can mitigate non‐radiative decay effectively. The shell plays an improtant role in passivating lattice defects on core surface, thereby enhancing the luminescence of core material. The connected AMD3100 was fabricated with the probes to bind the surface receptor CXCR4, enabling molecular‐level identification of tumors.

The lanthanide‐doped probes based on photoluminescence offer the benefits of rapid feedback and enhanced imaging resolution.^[^ ^24^ ^]^ Due to its distinctive NIR fluorescence property, the trivalent rare earth ion Nd‐doped probe has the capability to visualize and delineate tumors. In contrast to the NIR fluorescent dye ICG, RENPs show a sharp decrease in fluorescence within 15 minutes under continuous 808 nm laser irradiation, demonstrating greater stability, with minimal fluorescence quenching. Furthermore, it has been reported that ICG, a photosensitive NIR dye, is easily to be quenched upon laser exposure or long time storage in darkness.^[^ ^25^ ^]^ Conversely, RENPs have the ability to maintain their fluorescence after being store for more than six months under the same condition. These findings collectively suggest that the RENPs, serving as NIR luminescent materials, possess notably photostability.

Noteworthily, RENPs can visualize the metastatic breast cancer in real time accurately, so as to delineate a precise outline of tumors and guide targeted radiotherapy. In this study, the novel synthesized probe RENPs can be specifically enriched in tumor cells with highly expressed CXCR4, especially in TNBC.^[^ ^26^ ^]^ Many CXCR4 targeted drugs such as monoclonal antibodies, peptides, and small molecular inhibitors have been developed. For example, Delphine Séhédic et al. applied CXCR4 antibody clone 12G5 and Mahmoud S Alghamri et al. utilized CXCR4 inhibitor AMD3100 for glioma immunotherapy, showing a highly specific targeting the glioblastoma.^[^ ^27^ ^]^ In accordance with the aforementioned studies, our results suggest that CXCR4 might represent a promising target for molecular imaging in TNBC.

As reported previously, high atomic number lanthanide elements such as Au, Bi, and Lu, can effectively absorb X‐ray energy^[^ ^28^ ^]^ and promote tumor cell apoptosis to enhanced radiosensitivity.^[^ ^29^ ^]^ Radiosensitizers NBTXR3 containing Hf and AGuIX particles containing Gd have been evaluated in the clinical practice.^[^ ^30^ ^]^ Jeroen G. et al. found that ^177^Lu‐labelled star polymers significantly enhanced RT therapeutic efficacy in colon cancers.^[^ ^31^ ^]^ Our study found that the proliferation of tumor cells was significantly inhibited when combined RENPs and RT. In addition, combination of RENPs and RT induced intracellular ROS generation, leading to a significantly increase in proportion of apoptosis cells. Collectively, our findings indicated that lanthanide‐based Lu RENPs have the potential to act as radiosensitizers. In comparison to ^177^Lu‐labelled star polymers, the non‐radioactive Lu in RENPs enables accurate preoperative guidance for radiation therapy without causing harm to healthy tissues. In the future, the exploration of radiation sensitizer should combine different radiation doses and frequency in clinical practice to achieve better radiation therapeutic effect and minimize toxicity.

Immunosuppressive TME often leads to the immune escape in tumors and hinders antitumor immune response.^[^ ^32^ ^]^ Although RT can induce ICD and re‐activate antitumor immunity, RT‐induced systemic immune responses are too weak to meet clinical needs.^[^ ^33^ ^]^ To enhance the immune‐modulating effects of radiation, Huang et al. applied nanoscale coordination polymers to induce ICD by amplifying RT‐mediated oxidative stress.^[^ ^34^ ^]^ In our study, the RENPs synergized atezolizumab's effects, in particular in combination with RT. Importantly, the proportion of tumor killer CD8^+^ (TC or CTL cells) T cells was elevated to 56.94% and CD4^+^ T cells’ proportion has increased to 10.39% through radiosensitization in distant tumor. Correspondingly, cytokines (such as IL‐6, IFN‐γ) secreted by CD4^+^ T cells were obviously and robustly detected in radiosensitizer group, resulting in further activation of CTL cells and induction of macrophage antigen presentation. Besides, mature DCs secreted large amounts of cytokines including TNF‐α and IL‐12p70^[^ ^35^ ^]^ in radiosensitizer group, and significantly contributed to the inflammatory reaction. Meantime, IHC analysis showed the increase of PD‐L1 after the treatment of combination therapy of radiosensitization drugs, RT, and ICT, indicating a better anti‐PD‐L1 ICT efficacy by elevating PD‐L1 expression. The effectiveness of radioimmunotherapy in vivo clearly demonstrated RT‐induced immune activation, including DCs maturation, TILs recruitment and cell factors release, could be further amplified by RENPs in TNBC.

However, the limitation of this study is the utilization of murine breast cancer cell line xenograft model to activate immune system, and the anti‐tumor immunity was observed only in the mice. Thus, in the future, the patient‐derived tumor xenograft (PDX) models can be used to mimic human immune system and it's better to evaluate more accurate responses of the human immune system and TME by PDX models.

## 4 Conclusion

This study focuses on the development of lutetium‐doped lanthanide rare earth nanoparticles for radiation sensitization, incorporating an amphiphilic element and a CXCR4 targeting element AMD3100. Following successful synthesis and characterization of the probe, safety verification and targeting studies were conducted at cellular and animal levels. Additionally, the radiation sensitization properties of the probe were evaluated both in vitro and in vivo. Subsequently, radioimmunotherapy was implemented in small animal models to investigate the immune activation status of tumors and confirm the synergistic effects of RT and ICT.

In summary, the targeted multifunctional nanoprobe has been developed for imaging‐guided tumor delineation, radiosensitization and activation of immunity post‐radiation. This innovative RENP may hold significant promise for enhancing the effectiveness of radiotherapy and immunotherapy in TNBC.

## 5 Experimental Section

### Reagents

All reagents were used directly without further purification. Neodymium acetate (Nd(CH_3_COO)_3_, 99.99%), Ytterbium acetate (Yb(CH_3_COO)_3_, 99.99%), Lutetium acetate (Lu(CH_3_COO)_3_, 99.99%), 1‐octadecene (ODE, 90%) and oleic acid (OA, 90%) were purchased from Sigma‐Aldrich. NaOH (99.9%) and NH_4_F (99.99%) was purchased from the Shanghai Aladdin Bio‐Chem Technology. Anhydrous ethanol, cyclohexane, methanol and DMSO were purchased from Shanghai Sinopharm Chemical Reagent. Phosphate‐buffered saline (PBS) was acquired from Wuhan Procell Life Science & Technology. RPMI 1640 cell culture medium, trypsin‐EDTA and fetal bovine serum (FBS) were acquired from Gibco. Cell Counting kit‐8 (CCK‐8), ROS assay kits, and 4,6‐diamino‐2‐phenylindole (DAPI) were acquired from Beyotime. Anti‐CD3‐FITC, anti‐CD4‐APC, anti‐CD8‐PE antibodies for flow cytometry were purchased from BD Pharmingen. Anti‐PD‐L1 antibody atezolizumab for use in vivo was obtained from MCE. ELISA kits for IL‐6, TNF‐α, IL‐12p70, and IFN‐γ were purchased from MultiSciences.

### Cell Lines and Animals

TNBC cell line 4T1 and normal epithelial cell line MCF‐10A were acquired from the American Type Culture Collection (Rockvillle, USA). The cells were cultured in different medium according to vender recommendations. Female BALB/c mice (6‐8 weeks) were purchased from the Experimental Animal Center, Xiamen University. All animal studies in our research were approved by the Institutional Ethical Committee of Animal Experimentation, Xiamen University (The ethics approval number: No. XMULAC20180037).

### Synthesis of RENPs

*Synthesis of Core Structure NaNdF_4_*: *Yb*. Nd(CH_3_COO)_3_ (0.85 mmol), Yb(CH_3_COO)_3_ (0.15 mmol), OA (6 mL), and ODE (15 mL) was mixed and heated to 120 °C for 20 min in the vacuum. After the powder were dissolved, the mixture was cooled to 50 °C. Then 4 mmol of NH_4_F and 2.5 mmol of NaOH were added and heated to 100 °C for 30 min. After removing methanol in the mixture, it was subsequently heated to 300 °C for 90 min under argon gas. After high temperature reaction, the product was cooled to room temperature. Primary product was first collected by centrifugation (8000 rpm) with the introduction of ethanol. After washing 3 times, the primary product was dispersed in 5 mL of cyclohexane and stored at room temperature for further coating.

*Synthesis of Core‐Shell Structure NaNdF_4_*: *Yb@NaLuF_4_*. A mixture of Lu(CH_3_COO)_3_ (1 mmol), OA (6 mL), and ODE (15 mL) was added to capsule core structure NaNdF_4_:Yb. The procedure was almost the same as above. The only difference is the primary product was added in as well when adding NH_4_F and NaOH.

*Surface Modifications of NaNdF_4_:Yb@NaLuF_4_*. The NaNdF_4_:Yb@NaLuF_4_ (0.1 mmol) and DSPE‐PEG_2000_‐COOH (20 mg) were dissolved in chloroform (10 mL). Then, chloroform was evaporated slowly in a fume cupboard to obtain amino lipids modified RENPs (RENPs‐control). Subsequently, the product was dispersed in deionized water (DW) and excess DSPE‐PEG_2000_‐COOH were removed by ultra‐centrifugation (15,000 rpm).

*Synthesis of RENPs*. RENPs was first dissolved in 2‐(N‐Morpholino) ethanesulfonic acid (MES) solution (50 nM, pH 5.0 – 6.0), and then N‐Hydroxysuccinimide (NHS, 15 mg) and N‐(3‐Dimethylaminopropyl)‐N′‐ ethylcarbodiimide hydrochloride (EDC, 10 mg) were added and stirred gently for 2 h. Subsequently, AMD3100 (300 µg) were added into the above solution under continuously stirring for 12 h. The AMD3100 functionalized RENPs (RENPs) were purified by ultra‐centrifugation (15 000 rpm) with water and were finally dispersed in DW.

*Characterization of RENPs*. Transmission electron microscopy (TEM) images and FEI Talos F200s TEM mapping images were captured to investigate the morphology and elemental distribution of RENPs. Size distribution and zeta potential of RENPs were recorded by DLS. Luminescence spectra were determined using an FLS920 spectrometer. Absorption spectra were measured using a UV–vis spectrophotometer. Flow‐cytometric analysis was conducted using CytoFLEX LX (Beckman, USA).

### Cell Viability Assay

Cell viability was measured by CCK‐8 assay. 4T1 cells were seeded in a 96well plate at a density of 1 × 10^3^ per well and were treated with different concentrations of RENPs‐control and RENPs. Cells in treatment groups that received radiation were exposed to X‐ray in different doses. The SpectraMax M4 microplate reader was used to examine the absorbance in different groups. Then data was analyzed by Prism v9.5.0 software.

### Target of RENPs In Vitro

After 4T1 cells were seeded into 12 well plates, targeted and untargeted RENPs‐Rho were added by different concentrations (50, 100, 200 µg mL^−1^) at different time points (1, 3, 6 h). Treated cells were washed and analyzed by flow cytometer to measure the average fluorescence intensity (MFI). In addition, we use cell crawling slice images to detect cellular uptake of RENPs. First, 4T1 cells were cultured in a 12 well plate containing cell slides overnight. Then, the culture medium was replaced with a fresh medium containing targeted RENPs and untargeted RENPs‐control in different concentrations (50, 100, 200 µg mL^−1^) at different time points (1, 3, 6 h). The cell crawling slides were subsequently stained with DAPI staining solution and the images were detected by Nikon AI‐MP microscope.

### Colony Formation Assay

Colony forming ability was assessed to evaluate the growth potential of cancer cells after different treatments. A total of 1,000 4T1 cells well^−1^ were seeded into a 6 well plate. Crystal violet dye was used to count proliferative cells after 2 weeks of growth. These data were analyzed with ImageJ and are presented as colony number compared with untreated wells.

### ROS Detection

DCFH‐DA assay kit was used to detect intracellular ROS generation. The cell crawling slices were made as the procedure mentioned above. Cells in the RENPs groups were incubated with 50 µg mL^−1^ drugs and RT groups were exposed to 6 Gy X‐ray irradiation. Cell slides (FITC channels) were taken under Nikon AI‐MP microscope to observe ROS fluorescence signals.

### Apoptosis Assay

Annexin‐V FITC apoptosis detection kit was used to measure cell death. Cells were seeded at a density of 1 × 10^6^ per well in a 6 well plate and were incubated with RENPs or treated with RT. After 24 h of treatment, cells were stained by propidium iodide and Annexin V, which were later analyzed by FACS Canto II flow cytometer.

### Radiosensitization of RENPs In Vivo

For the unilateral tumor model, 1 × 10^6^ cells resuspended in PBS were injected subcutaneously into the right flank of female BALB/c mice. When the average tumor volume reached ≈50 mm^3^, the mice were randomly divided into four groups treated with PBS, RENPs (5 mg kg^−1^). All reagents were injected intravenously at tail vein, and body weight and tumor volume were monitored every two days. Tumor volume was calculated as length × width^2^/2. On the 20th day, all mice were sacrificed and the major organs, spleen and serum were collected for analysis.

### Radioimmunotherapy Combined with RENPs In Vivo

For the bilateral tumor model, mice were injected with 4T1 cells into the left (5 × 10^5^) and right (1 × 10^6^) flank regions on the same day. The right site was considered the primary tumor for direct treatment, and the left site was considered a distal tumor. When the primary tumor volume reached ≈50 mm^3^, the mice were randomly divided into five groups treated with RENPs, RT, and ICT. Anti‐PD‐L1 drug atezolizumab was injected intraperitoneally at 100 µg per mouse on days 2, 4, and 6, whereas RENPs were injected through tail vein in 100 µL on days 1, 3, and 5. After 3 h intravenous injection, mice were irradiated with 6 Gy X‐ray irradiation. Individual tumor growth at both sides and body weight were carefully monitored. On the 20th day, all mice were sacrificed and the major organs, spleen, and serum were collected for analysis.

### Immune Response Analysis

For CD8^+^ and CD4^+^ T cell analyses in spleen and tumor tissues, cell suspensions (spleens or tumor tissues) were stained with anti‐CD3‐FITC, anti‐CD4‐APC, and anti‐CD8‐PE, which were also analyzed by immunofluorescence staining. For cytokine testing, serum was obtained from eyeballs isolated from the mice after therapy completion. IFN‐γ, TNF‐α, IL‐6, and IL‐12p70 concentrations were determined using ELISA kits per the manufacturer's instruction.

### Toxicity of RENPs In Vivo

*Hemolysis Experiment*. After taking blood from BALB/c mouse, add 0.2 mL anticoagulant and wash it twice with PBS to remove the white blood cells and obtain 2% red blood cells. The physiological saline group is negative control, the DW group is positive control and add different concentration of RENPs. After 3 hours’ incubation, supernatant was measured by SpectraMax M4 microplate reader to determine its OD value at 450 nm and calculate the hemolysis. Then data was analyzed by Prism v9.5.0 software.

*Weight and Histological Monitor In Vivo*. BALB/c mice (female, aged 6–8 weeks) were randomly divided into five groups (*n* = 5). RENPs (10 mg kg^−1^, 200µL) was injected intravenously on days 1, 3, 7, and 28. In control group, mice were injected with PBS. The weight of mice was monitored every 2 days. Blood was collected for blood routine and blood biochemical tests. Major organs (heart, liver, spleen, lung, and kidney) were collected for histological H&E analysis.

*Distribution of RENPs In Vivo*. BALB/c tumor bearing mice were sacrificed 3 h after injection of 10 mg kg^−1^ RENPs and important organs of the mice were collected. In vitro fluorescence images of the tissues and organs were captured using IVIS Lumina II imaging system and ROI were analyzed by Prism v9.5.0 software.

### Statistical Analysis

Data analyses were performed using Excel 2019 (Microsoft, USA) and Prism v9.5.0 (GraphPad, USA). The data were analyzed using Student's *t* test or one‐way analysis of variance (ANOVA). It is considered statistically significance when *P* < 0.05. All statistical analyses are shown in the figure legends.

## Conflict of Interest

The authors declare no conflict of interest.

## Supporting information

Supporting Information

## Data Availability

The data that support the findings of this study are available from the corresponding author upon reasonable request.
