# Supplementary material for: Rare Earth Nanoprobes for Targeted Delineation of Triple Negative Breast Cancer and Enhancement of Radioimmunotherapy
Source: Adv Sci (Weinh). 2024 May 22;11(29):2309992. doi: 10.1002/advs.202309992 (PMC11304243; doi:10.1002/advs.202309992)
Supplement: Supplementary file 1 — Supporting Information [file ADVS-11-2309992-s001.pdf]

ADVANCED SCIENCE
Open Access

Supporting Information

for *Adv. Sci.*, DOI 10.1002/advs.202309992

Rare Earth Nanoprobes for Targeted Delineation of Triple Negative Breast Cancer and Enhancement of Radioimmunotherapy

Zi-He Ming, Yong-Qu Zhang, Liang Song, Min Chen, Lin-Ling Lin, Yue-Yang He, Wan-Ling Liu, Yuan-Yuan Zhu, Yun Zhang and Guo-Jun Zhang**

Supporting Information

Additional characterization data for RENPs and bioapplication of RENPs are included in Figures S1−S11.

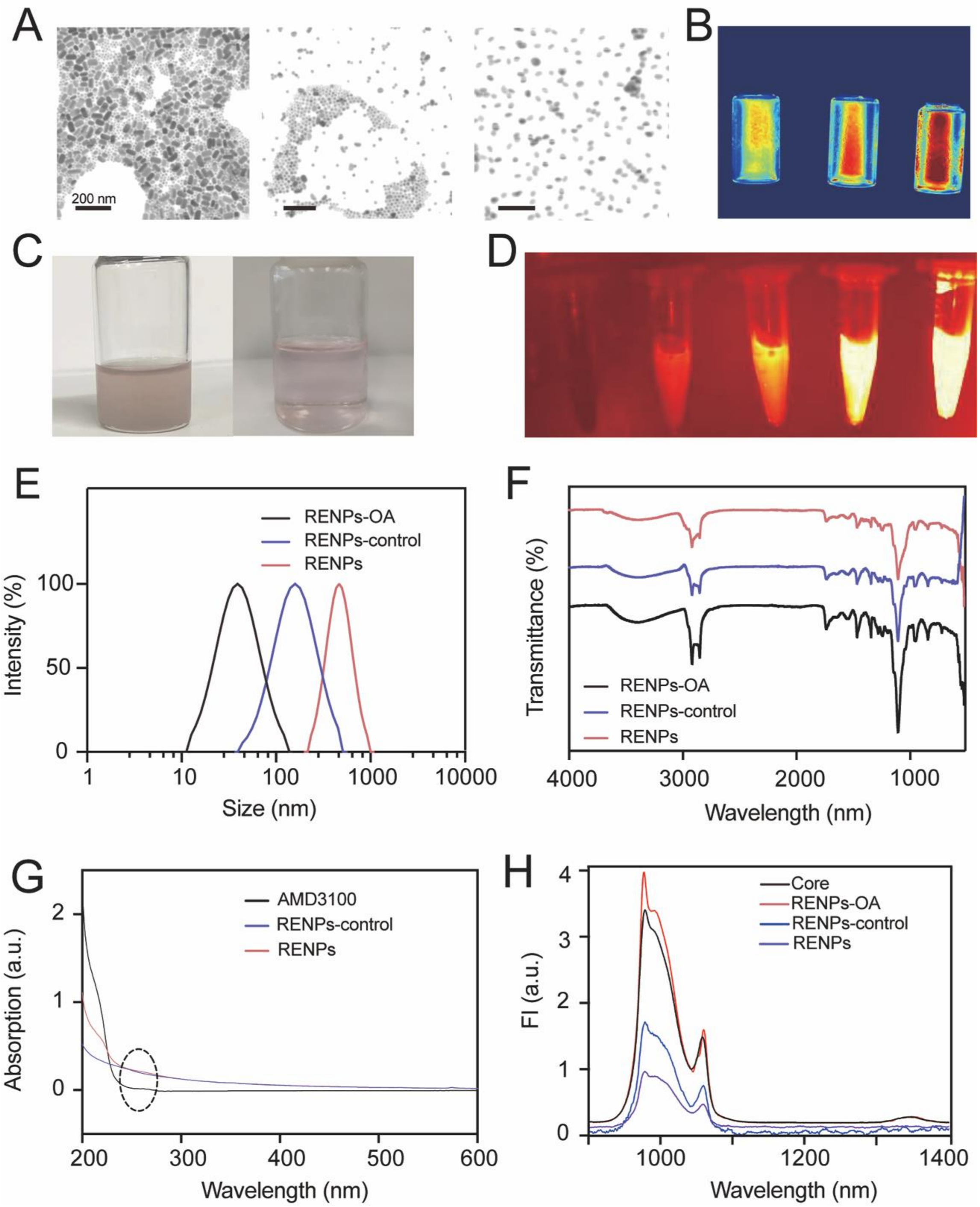

Figure S1. Synthesis and characterization of probes. (A) The TEM image of different ratio of Yb in RENPs, from left to right is 5%, 10%, and 15%. **(B)** The NIR-II imaging of different ratio of Yb in RENPs, from left to right is 5%, 10%, and 15%. **(C)** Photographs of core (NaNdF4:Yb) and core-shell (NaNdF4:Yb @ NaLuF4) in cyclohexane. **(D)** The NIR imaging of different concentration of RENPs, from left to right is 0, 10, 25, 50 and 100 μg/mL. **(E)**

DLS analysis of RENPs-OA, RENPs-control, and RENPs. **(F)** Fourier infrared spectroscopy of RENPs-OA, RENPs- control and RENPs. **(G)** UV-vis spectra of AMD3100, RENPs-control and RENPs. **(H)** Fluorescence emission spectra of various nanoparticles including core, RENPs, RENPs-control, and the final RENPs.

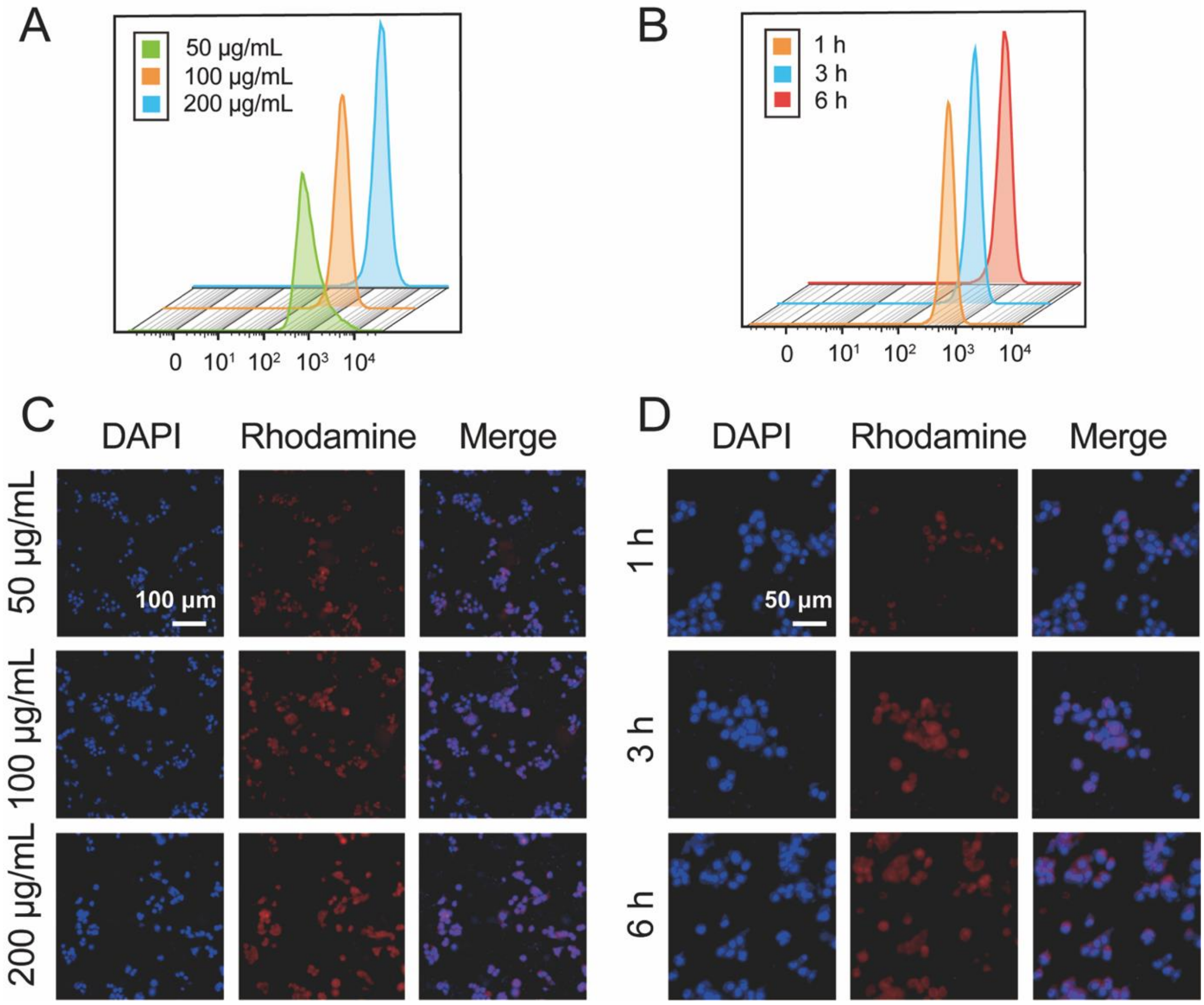

Figure S2. Uptake of RENPs under different incubation time and concentration. (A, B) Flow cytometry analysis of 4T1 cells after incubated with RENPs in different concentration and times. **(C, D)** Fluorescence microscope scanning of cell crawling imaging in 4T1 cells after incubated with RENPs in different concentrations and times.

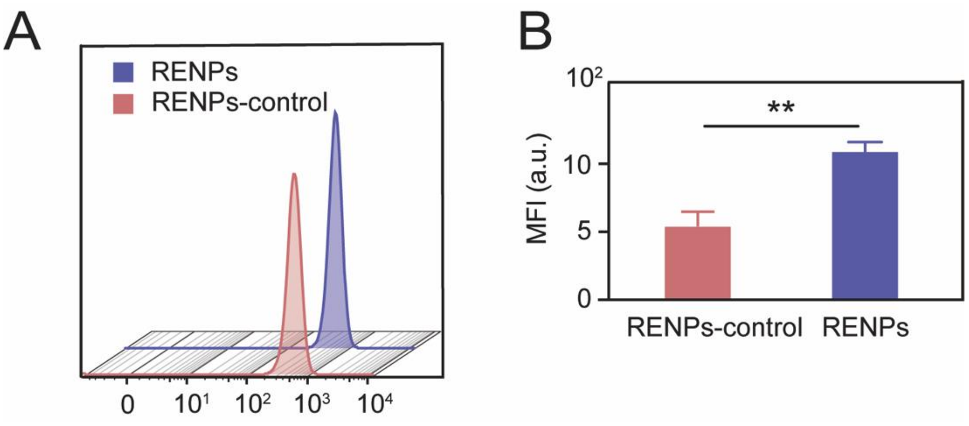

Figure S3. Target effect of RENPs. (A) Flow cytometry of 4T1 cells incubated with RENPs-control / RENPs and **(B)** corresponding quantified of MFI (n=3). ** $P < 0.01$.

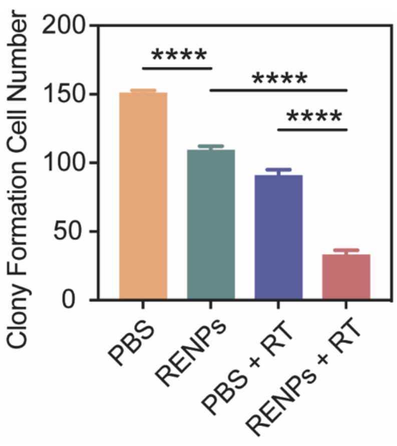

Figure S4. Quantitative analysis of 4T1 colony formation assays. 4T1 cells were treated with PBS and RENPs before irradiation (6 Gy). **** $P < 0.0001$.

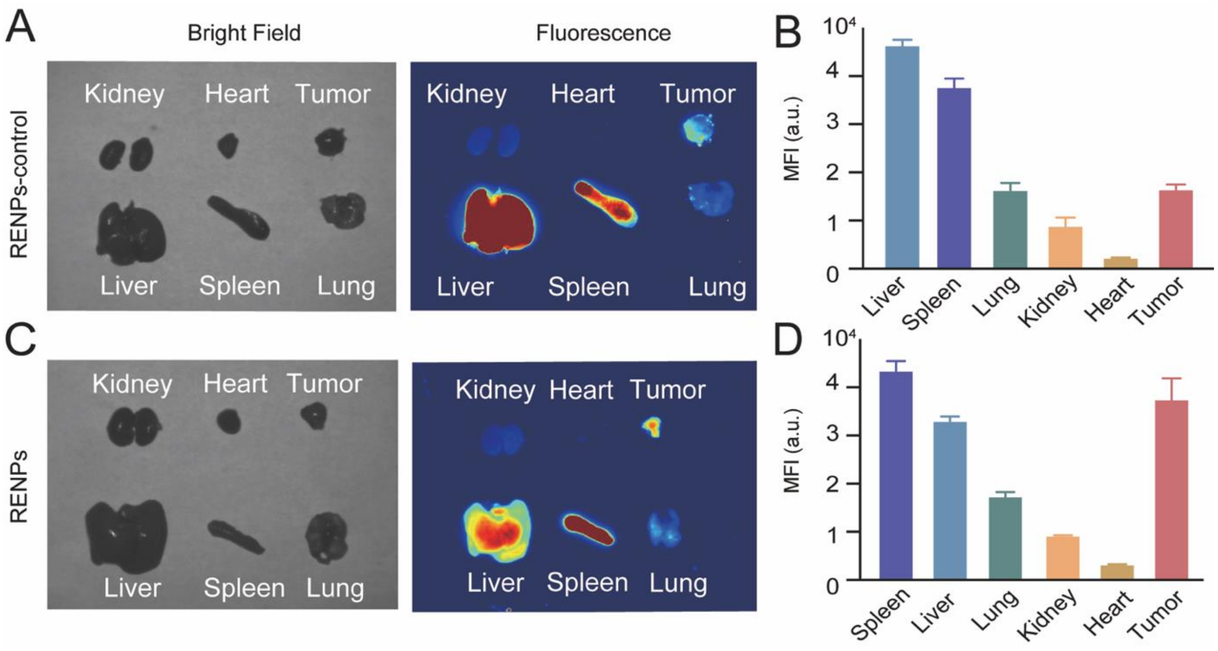

Figure S5. Imaging of excised organ and tumor tissues. (A) Bright and NIR fluorescence images of organs and tumors collected after injected with RENPs-control and **(B)** corresponding semiquantitative analysis of mean sample fluorescence intensity (n = 3). **(C)** Bright and NIR fluorescence images of organs and tumors collected after injected with RENPs and **(D)** corresponding semiquantitative analysis of mean sample fluorescence intensity (n = 3).

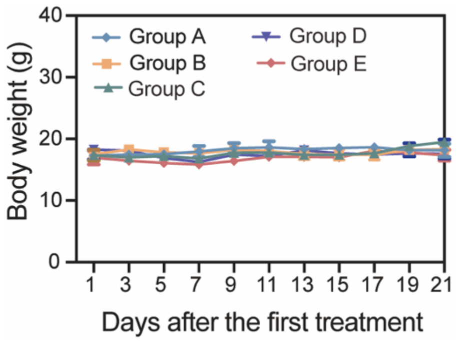

Figure S6. Body weight of mice after different treatments.

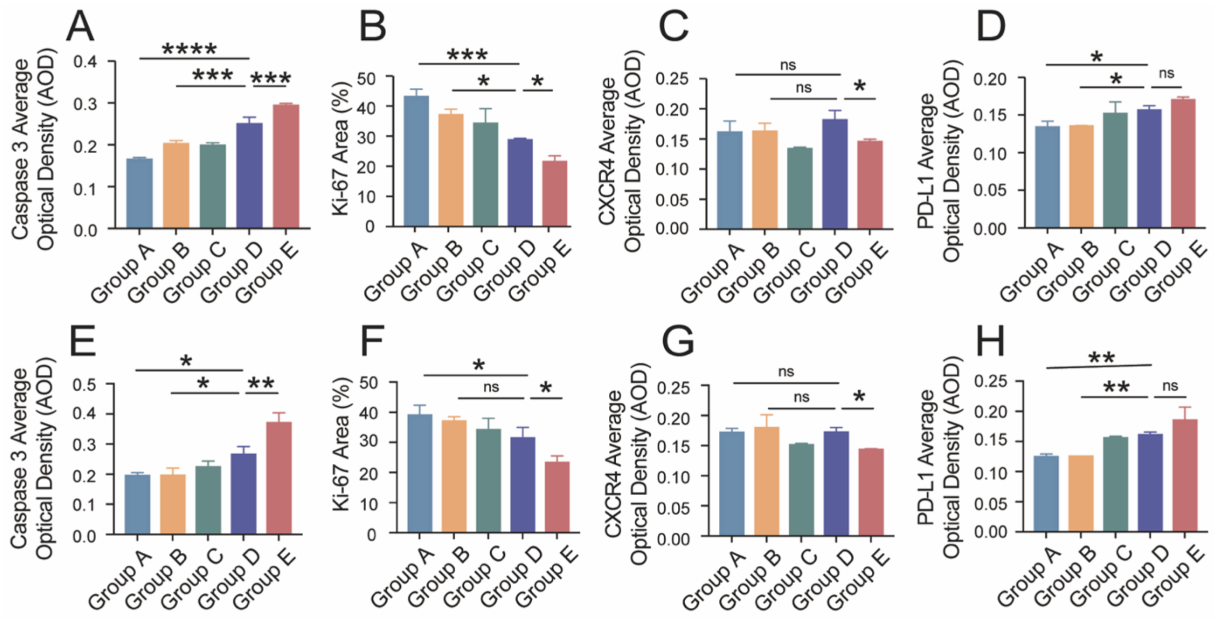

Figure S7. Quantified analysis of IHC.

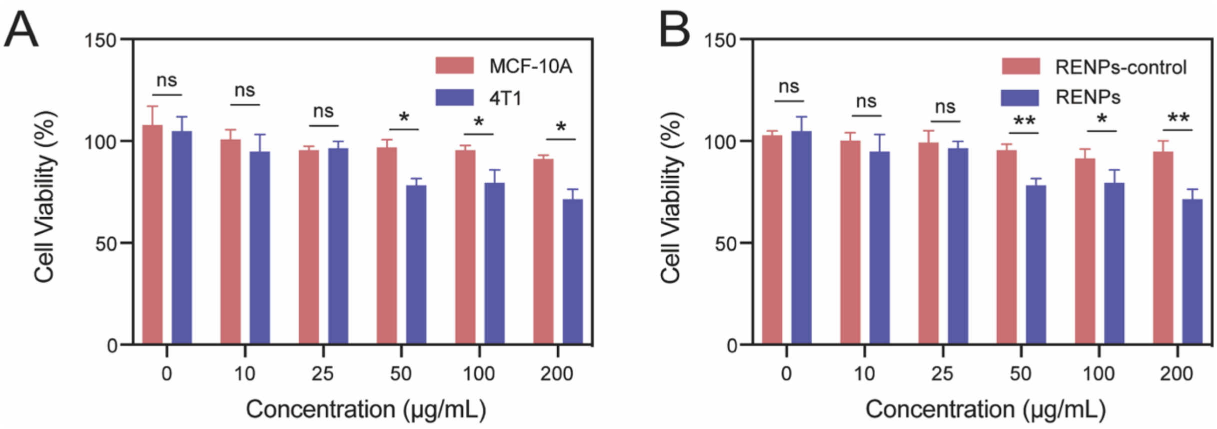

Figure S8. Cell viability of different cells incubated with different probes. (A) Viability of 4T1 and MCF-10A cells after incubated with RENPs. **(B)** Viability of 4T1 cells incubated with RENPs-control / RENPs.

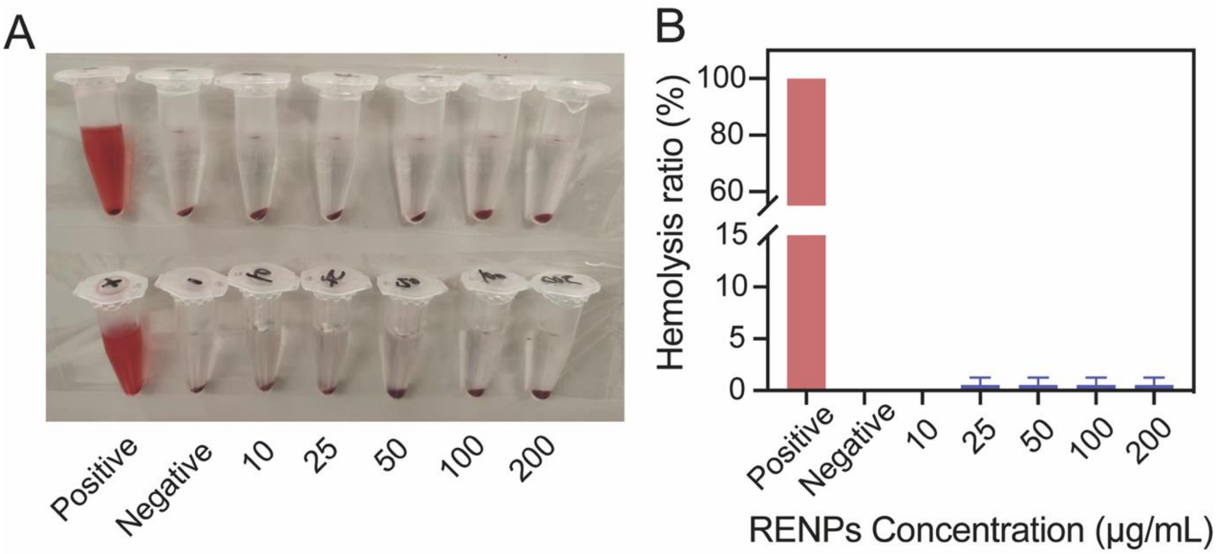

Figure S9. Hemolytic property of probes. (A) Images of hemolysis test and **(B)** quantified of hemolysis ratio.

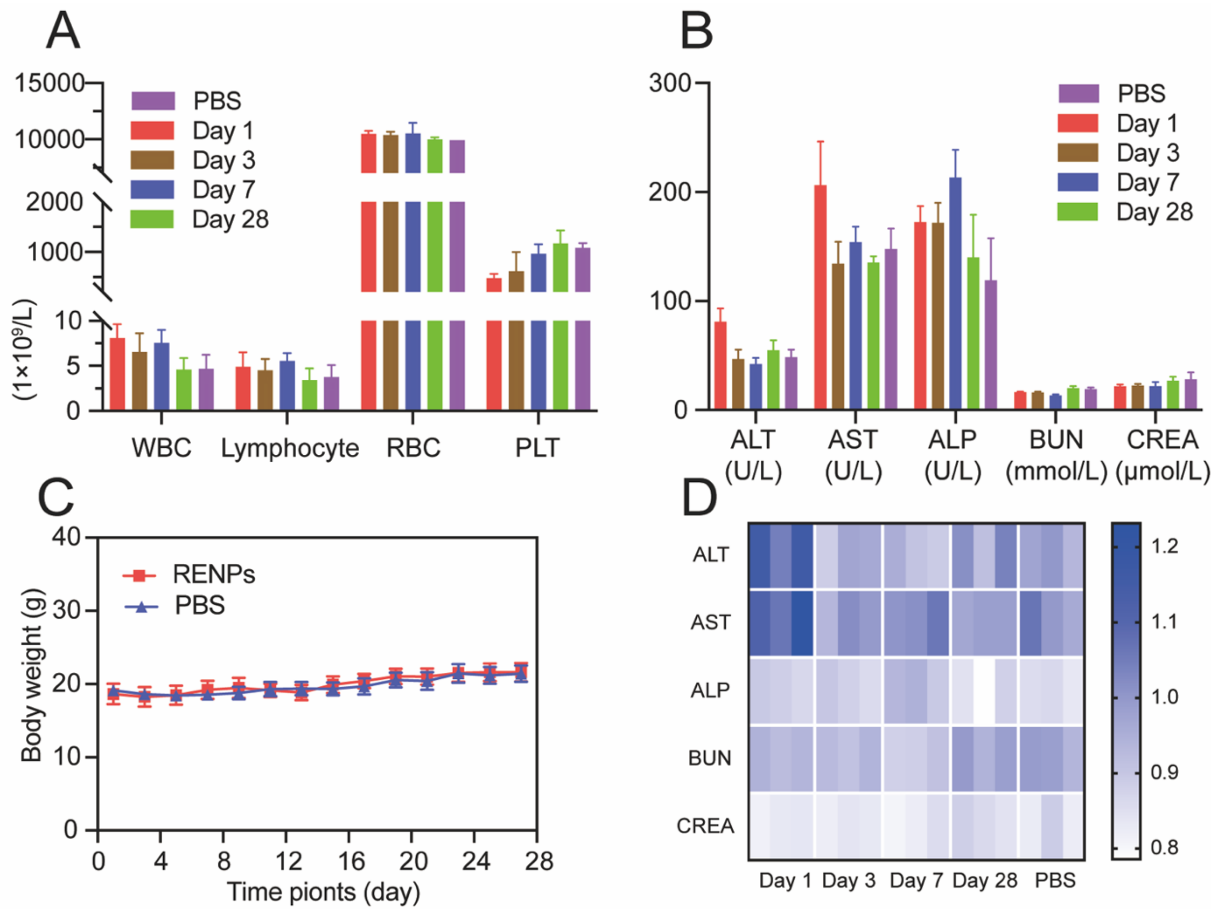

Figure S10. Biosafety of mice injected with probes. Assessment of blood routine **(A)** for 28 days after injection of RENPs and PBS, and the blood serum biochemistry parameters **(B)** as well as weight **(C)** at different time points to determine the effect on blood cells, liver function and kidney function (n = 3 for each group). **(D)** The color variance represents the biochemical analysis data.

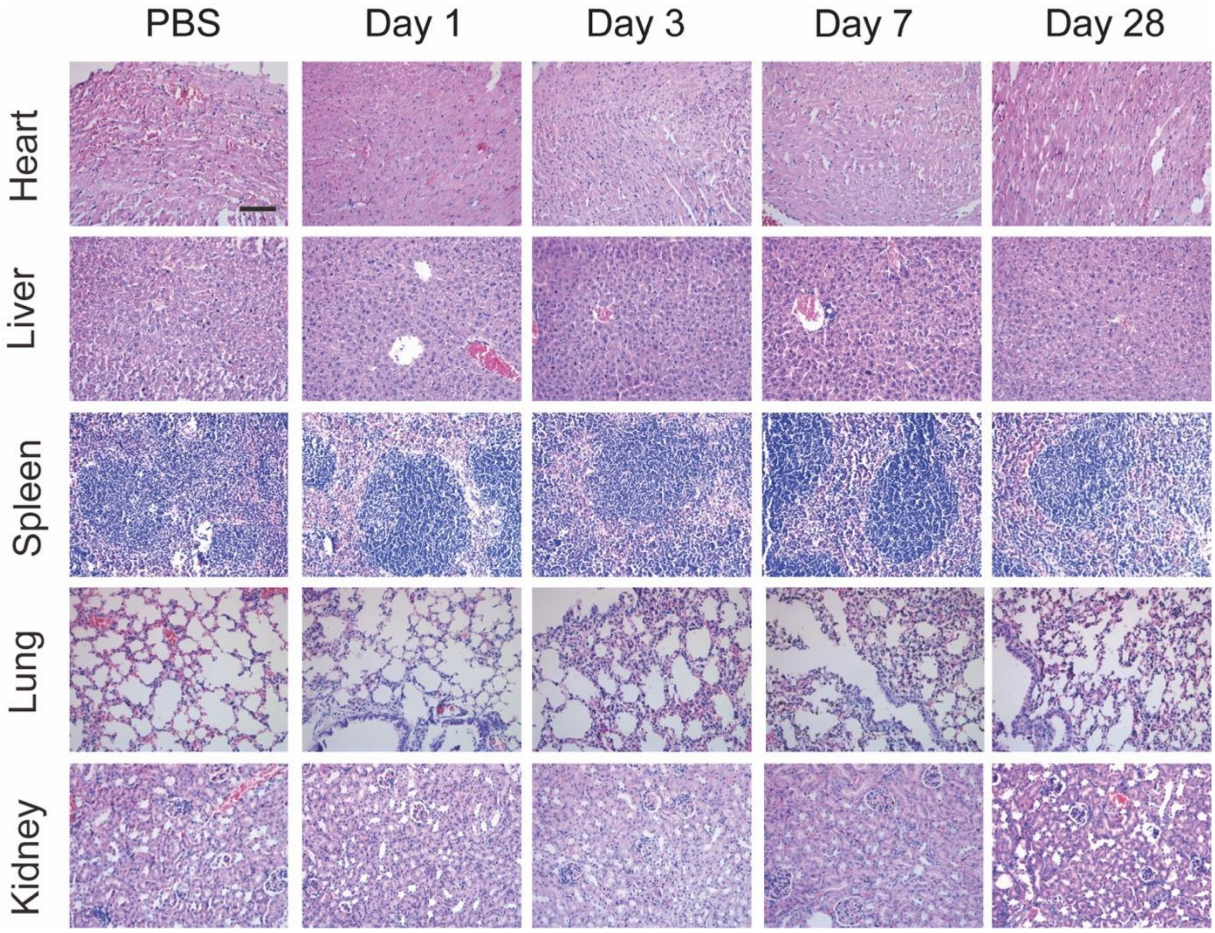

Figure S11. Hematoxylin and eosin staining of major organs (heart, liver, spleen, lung and kidney) from BALB/c mice at 28 days after intravenous injection of PBS and different time points after intravenous injection of RENPs (n = 3 for each group). Scale bar, 100 μm.

Supporting Information is available from the Wiley Online Library or from the author.
